# Analyzing the equity of public primary care provision in Kenya: variation in facility characteristics by local poverty level

**DOI:** 10.1186/1475-9276-11-75

**Published:** 2012-12-13

**Authors:** Mitsuru Toda, Antony Opwora, Evelyn Waweru, Abdisalan Noor, Tansy Edwards, Greg Fegan, Catherine Molyneux, Catherine Goodman

**Affiliations:** 1 Kenya Medical Research Institute, Wellcome Trust Research Programme, P.O. Box 43640, Nairobi, Kenya; 2 Malaria Public Health and Epidemiology Group, Centre for Geographic Medicine, Kenya Medical Research Institute - Wellcome Trust Research Programme, P.O. Box 43640, Nairobi, Kenya; 3 Centre for Tropical Medicine, Nuffield Department of Clinical Medicine, University of Oxford, CCVTM, Oxford, OX3 7LJ, UK; 4 MRC Tropical Epidemiology Group, London School of Hygiene & Tropical Medicine, Keppel St, London, WC1E 7HT, UK; 5 Department for Global Health and Development, London School of Hygiene & Tropical Medicine, Keppel St, London, WC1E 7HT, UK

**Keywords:** Health equity, Facility characteristics, Access, Primary care, Poverty, Kenya

## Abstract

**Introduction:**

Equitable access to health care is a key health systems goal, and is a particular concern in low-income countries. In Kenya, public facilities are an important resource for the poor, but little is known on the equity of service provision. This paper assesses whether poorer areas have poorer health services by investigating associations between public facility characteristics and the poverty level of the area in which the facility is located.

**Methods:**

Data on facility characteristics were collected from a nationally representative sample of public health centers and dispensaries across all 8 provinces in Kenya. A two-stage cluster randomized sampling process was used to select facilities. Univariate associations between facility characteristics and socioeconomic status (SES) of the area in which the facility was located were assessed using chi-squared tests, equity ratios and concentration indices. Indirectly standardized concentration indices were used to assess the influence of SES on facility inputs and service availability while controlling for facility type, province, and remoteness.

**Results:**

For most indicators, we found no indication of variation by SES. The clear exceptions were electricity and laboratory services which showed evidence of pro-rich inequalities, with equity ratios of 3.16 and 3.43, concentration indices of 0.09 (p<0.01) and 0.05 (p=0.01), and indirectly standardized concentration ratios of 0.07 (p<0.01) and 0.05 (p=0.01). There were also some indications of pro-rich inequalities for availability of drugs and qualified staff. The lack of evidence of inequality for other indicators does not imply that availability of inputs and services was invariably high; for example, while availability was close to 90% for water supply and family planning services, under half of facilities offered delivery services or outreach.

**Conclusions:**

The paper shows how local area poverty data can be combined with national health facility surveys, providing a tool for policy makers to assess the equity of input and service availability. There was little evidence of inequalities for most inputs and services, with the clear exceptions of electricity and laboratory services. However, efforts are required to improve the availability of key inputs and services across public facilities in all areas, regardless of SES.

## Introduction

Equitable access to health care is emphasized as a key health systems goal [1-4]. Governments have attempted to address this goal through a range of measures including reducing user fees, implementing social insurance schemes, and enhancing health care infrastructure at the periphery [4]. Despite these interventions and policy shifts, studies have shown that the benefits of public health care expenditure are often disproportionally obtained by the relatively better off [1,4-7]. The poor contribute a larger proportion of their income to health care than the rich [8], and poorer populations frequently receive poorer quality of care [9]. For example, tax-financed health services may benefit those in the urban middle and upper classes disproportionately even if the services are intended for the poor, because the relatively better off are located close to better services, and are more likely to use them because of their superior resources and connections [1]. Benefit-incidence analyses have shown that the majority of public health budgets are pro-wealthy [5,6], and that regardless of source of financing, proximity, or whether care is delivered through the public or private sector, the better-off are more likely to get access [4,7].

In Kenya, equitable access to health services is emphasized strongly in national policy documents [10-13]. The National Health Sector Strategic Plan (NHSSP II 2005–2010), for example, explicitly states that increasing equitable access to health services is one of its main goals [13]. The majority of the population live in rural areas (67.7%), and nearly half (46%) live below the poverty line [14,15]. Public primary health care facilities, such as health centers and dispensaries, are a particularly important source of services for the poor [16]. Chuma et al. have shown that the poorest population quintile in Kenya have the highest health needs but receive the lowest share of total health systems benefits [17]. Further research is therefore warranted to understand the cause of such inequalities. In particular, little is known about the equity of primary health care services provision.

This paper aims to assess whether people in poorer areas of Kenya have poorer health care services by investigating associations between public primary care facility characteristics and the poverty level of the area in which the facility is located.

## Methods

### Study design

Data on facility characteristics were collected as part of a national primary care health facility survey in Kenya. A nationally representative sample of health centers and dispensaries was selected across all 8 provinces, stratified by facility type and by municipal/non-municipal area. A two-stage cluster randomized sampling process was used. First, we randomly selected 3 districts per province in the 7 provinces of North Eastern, Eastern, Central, Rift Valley, Western, Nyanza and Coast (21 districts in total)^a^. In addition, we randomly selected one district from each of the 3 municipal areas: Nairobi, Mombasa and Kisumu.

Our second step was to randomly select 7 health centres and 7 dispensaries per district. Dispensaries are the lowest level of outpatient health facility in Kenya, while health centres are slightly larger and may have some inpatient beds. Within each selected district, our sampling frame included all government-owned health centers and dispensaries open to the general public that were officially recognized by the government, operational, and had at least one qualified health worker at the time of the survey. This included Ministry of Public Health and Sanitation (MOPHS) and local authority facilities but excluded prison and army facilities. We randomly selected 7 facilities of each facility type (health center and dispensary) per district. In districts with less than 8 facilities of a given type, we surveyed all relevant facilities in that district.

Prior to data collection in July 2010, we confirmed with the relevant District Health Management Teams (DHMTs) that the selected facilities were operational. Twenty-four (4.6%) of the facilities were excluded because they were not operational or were temporarily closed. If a facility was not operational at the time of the survey, we replaced the facility where possible with the next available facility on our list of randomly selected facilities. In total, we collected data from 248 facilities: 144 non-municipal dispensaries, 65 non-municipal health centers, 21 municipal dispensaries and 18 municipal health centers [18]^b^.

### Data collection on facility characteristics

Data were collected between July and September 2010. At each facility, we conducted structured interviews with the health worker in-charge of the facility. The survey tool drew on the Kenya Service Provision Assessment 2004, and included questions on availability of facility inputs such as infrastructure, equipment, staffing and commodities, and availability of services provided. The data collection instrument was piloted extensively in rural and urban districts prior to administration.

Data were entered at the point of data collection into mini-laptops by the field interviewers and were transferred every day electronically into a central database. Data were merged using MS Access (Microsoft, USA) and then imported into Stata version 11 (StataCorp, USA) for analyses. Data were checked and cleaned using Stata do-files and any questions returned to the field staff.

Informed oral consent was obtained for all interviews and the ethical review committees of Kenya Medical Research Institute (KEMRI) and the London School of Hygiene and Tropical Medicine (LSHTM) approved the study. Interviewee names were not recorded to protect confidentiality.

### Selection of facility characteristics for analysis

We identified two sets of facility characteristics related to facility inputs and service availability. Under facility inputs, we measured availability of functioning equipment, functioning infrastructure, and commodities in stock. In addition, we looked at staff assigned to the facility (we did not assess the presence of staff on the day of the survey) (Table 1). On equipment, we measured whether the facility had at least 15 out of 18 key items available and functioning. Under infrastructure, we measured availability of any electricity and any water supply. Under commodities, we measured three variables: availability of all drugs on the MOPHS tracer list [19], all items in a set of key family planning commodities, and all items in a set of key vaccines as reported by the health worker in-charge. On availability of staff, we created two variables: whether a facility had at least 4 staff with medically related qualifications, and whether they had at least 2 support staff.

**Table 1:** Facility input variables: definitions and percent of facilities with inputs available

**Inputs available**	**Definition**	**Number of facilities with inputs available**	% **of facilities with inputs available** [**95**% **CI**]
Equipment & infrastructure			
Equipment	At least 15 out of the 18 key items available on the day of the survey	158	62.0 [53.2-70.1]
N=248	[Any communication equipment (mobile phone, telephone or two-way radio); Any means of transport (bicycle, motorbike or own vehicle); Labour bed/ couch; BP machine; Stethoscope; Thermometer; Adult weighing scale; Baby weighing scale; Sharps box; Decontamination solution; Steriliser (cooker or stove); Disposable gloves; Dressing material (gauze and tape); Waste receptacle with lid and plastic liner; Hand washing soap; Supplies to mix ORS, cups and spoons; Refrigerator; Fridge thermometer]		
Electricity	Any electricity supply available	120	37.5 [22.1-56.0]
N=247			
Water	Any water supply available	222	87.2 [76.9- 93.3]
N=248	[Running piped, non-running piped, stored]		
Commodities			
Drugs	All drugs on tracer list in stock on the day of the survey	48	19.2 [12.9-27.6]
N=248	[Paracetamol tablets; Albendazole tablets; Amoxicillin Capsules; Amoxicillin Syrup; Co-trimoxazole tablets; Chlorphenamine (Piriton) tablets; Artemether-lumefantrine tablets; Hydrocortisone injection; Tetracycline eye ointment; Metronidazole (Flagyl) tablets; Clotrimazole cream; Injection Benzyl penicillin; Injection Gentamycin; ORS 500ml sachets]		
Family planning commodities	All essential family planning commodities in stock on the day of the survey	180	76.6 [68.7-83.0]
N=247	[Contraceptive pills; Emergency contraceptive pills; Depo-Provera injections; Male condoms]		
Vaccine commodities	All essential vaccines in stock on the day of the survey	199	77.5 [66.6-85.6]
N=247	[Tetanus toxoid; BCG; Measles; OPV; Pentavalent (DPT/ HepB/ Hib]		
Staff			
Qualified staff	At least 4 staff with medically related qualifications available	141	46.6 [37.4-56.1]
N=248	[e.g. clinical officer, registered nurse, enrolled nurse, pharmaceutical technician, lab technologist, lab technician, public health technician, public health officer, social worker, VCT counsellor, health education officer]		
Support staff	At least 2 support staff available	168	63.2 [55.3-70.6]
N=248	[e.g. cleaner, watchman, data clerk, clinical support staff, mixed category, administrative support, cashier, cook, and others]		

Note: BP = Blood Pressure; ORS = Oral Re-hydration Salts; BCG = Bacillus Calmette Guerin (TB vaccine); OPV = Oral Polio Vaccine; DPT = Diphtheria, Pertussis (Whooping cough) and Tetanus vaccine; HepB = Hepatitis B; Hib = Haemophilus influenzae type B vaccine; VCT = Voluntary Testing and Counseling.

We investigated the availability of the following services: laboratory, family planning, deliveries, Voluntary Counseling and Testing/ Provider Initiated Testing and Counseling (VCT/PICT), Prevention of Mother to Child Transmission (PMTCT), Anti-Retroviral Treatment (ART), Insecticide Treated Nets (ITNs), and any outreach services conducted in the past quarter (April – June 2010) (Table 2). We also assessed whether all childhood immunizations and all antenatal care were offered every weekday.

**Table 2:** Service availability variables: definitions and percent of facilities with services available

**Services available**	**Definition**	**Number of facilities with services available**	% **of facilities with services available**
			[**95**% **CI**]
Laboratory	Laboratory services available	115	38.1
N=246			[25.1-53.0]
Family planning	Family planning services offered	237	97.4
N=247			[93.3-99.0]
Delivery	Delivery services offered	143	49.0
N=247			[37.9-60.2]
VCT/PITC	VCT/PITC services offered	216	85.2
N=247			[77.3-90.7]
PMTCT	PMTCT services offered	219	88.1
N=240			[76.6-94.4]
ART	ART services offered	93	26.6
N=247			[18.2-37.0]
ITNs	ITNs available for distribution	194	84.4
N=247			[71.8-92.0]
Outreach	At least one outreach activity conducted in the past quarter (April – June 2010)	123	44.8
N=247			[32.1-58.1]
All childhood immunizations every weekday	All childhood immunization services available Monday to Friday	136	45.6
N=247			[25.3-67.5]
All antenatal care every weekday	All antenatal care services available Monday to Friday	203	74.6
N=246			[57.1-86.6]

Note: VCT = Voluntary Counseling and Testing; PITC = Provider Initiated Testing and Counseling; PMTCT = Prevention of Mother to Child Transmission; ART = Anti-Retroviral Treatment; ITNs = Insecticide Treated Nets.

### Measuring socioeconomic status (SES) of the local area for each facility

We used the proportion of the population above the poverty line in the location in which the sampled facility was located as a measure of socioeconomic status of the facility’s local area. The location is the second lowest administrative area (4^th^ level) in Kenya, containing a median population of 3,122 and a median area of 16km^2^[20]. We chose the location as the unit of analysis because it is the smallest area for which poverty data were available, and it provides a rough approximation of facility catchment area. However, it should be noted that the location does not necessarily correspond with the official MOPHS catchment area, or the area in which most facility users reside.

The most recent data available to assess poverty level at the location level were from 1997/99. The poverty line was estimated in 1997 prices at USD 21.2 and USD 45.3 (KES 1,239 and KES 2,648)^c^ per month for rural and urban households respectively [20]. The estimation of the proportion of households above the poverty line in each location was based on expenditure and consumption data from the 1997 Welfare Monitoring Survey (WMS)^d^ and the 1999 population and housing census. The census did not contain household expenditure data but, since the census and the WMS contained socio-economic variables such as household size, housing characteristics and availability of basic services, it was possible to statistically infer household expenditures for all censused households using regression analysis. On this basis, 52.9% of the rural population in Kenya lived under the poverty line and 49.2% of the urban population [21]. Sampled facilities in our survey were grouped into weighted SES quintiles. The cutoff points for the quintiles and the distribution of facilities by poverty level are described in Table 3.

**Table 3:** Definition of socioeconomic status (SES) quintiles, and number of sampled facilities falling into each quintile

**SES quintiles**	**Number of facilities**^**1**^	**Percent of households above the poverty line cut**-**offs**	
		**Range** (**min**-**max**)	**Mean**
Poorest 20%	68	17.8-36.9	31.1
2^nd^ quintile	65	37.0-43.9	39.9
3^rd^ quintile	30	44.0-51.0	47.6
4^th^ quintile	38	51.1-61.0	56.4
Least poor 20%	47	61.7-92.1	71.5

^1^ The number of facilities per quintile is not equal as sampling weights were used in calculating the quintile cut-offs.

### Data analysis

The analysis took into account the survey design by using the *svy* commands in Stata version 11 to adjust for clustering at the district and facility levels and stratification by facility type and municipal/non-municipal area. Differences in sampling probability across facility type and districts were allowed for using sampling weights.

Univariate associations between facility characteristics and socioeconomic status were assessed using chi-squared tests, equity ratios and concentration indices. The equity ratio for a given indicator is calculated as (percentage in least poor quintile/ percentage in poorest quintile), with a ratio greater than one implying pro-rich inequalities [22]. The concentration index ranges between −1 to +1 with zero indicating equality and a positive index indicating pro-rich inequalities [23]. Concentration indices and associated p-values, showing whether the index was significantly different from zero, were calculated in Stata [23-25]. Indirectly standardized concentration indices [23] were used to assess the influence of SES on facility inputs and service availability while controlling for facility type, province, and remoteness. The use of concentration indices for binary variables has been criticized because the index depends on the mean of the variable being analyzed, with the minimum and maximum possible values on the index changing accordingly. Nevertheless, they remain widely used and there is no consensus on an appropriate alternative [26-29].

Remoteness was measured using information on the distance between the sampled facility and 268 main towns, including 149 district headquarters. The definition of main towns was based on the Kenyan national census of 2009 [30]. The measure of remoteness was created using a straight-line calculation method in ArcGIS 9.2 (ESRI Inc., USA), which does not take into account the complex nature of terrains and transportation services. We grouped the remoteness indicator into three categories: near (<6km), middle, and far (>=31km (Table 4)). The cut-offs were based on empirical studies, which found that most facility users traveled 5-6km to access government facilities [31,32] and 30km for hospitals [33,34].

**Table 4:** Definition of remoteness categories, and number of sampled facilities falling into each category

**Remoteness categories**	**Number of facilities**	**Distance** (**km**) **from main town cut**-**offs**	
		**Range** (**min**-**max**)	**Mean**
Near	80	0-5.8	3.6
Average	140	6-30.6	13.5
Far	28	31-77.1	44.1

## Results

**Figure 1:**
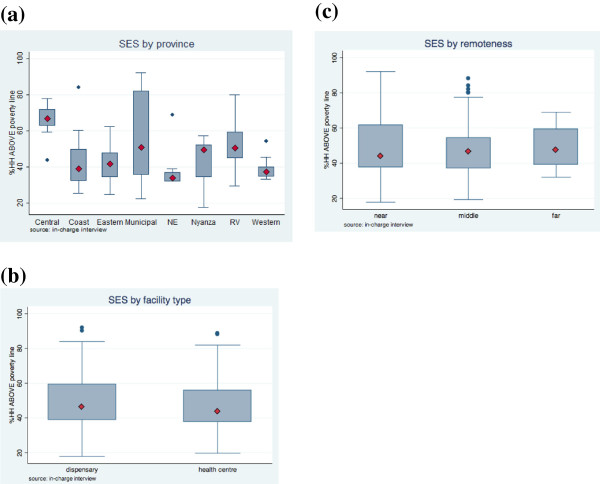
**Proportion of households above the poverty line by (a) province, (b) facility type, and (c) remoteness (Box plots represent 25th to 75th percentiles, diamonds represent medians, and whiskers represent minimum and maximum values).** Note: Municipal areas included districts of Kilindini (Coast province), Nairobi West (Nairobi province), and Kisumu East (Nyanza province). NE=North Eastern; RV=Rift Valley.

There was no significant variation in SES of the facility’s location by facility type, and remoteness (Table 5 and Figure 1). However, there were marked geographical variations in SES at the provincial level. For example, Central Province had the highest concentration of facilities in the least poor quintile, while North Eastern and Western Provinces had the highest concentration of facilities in the 2^nd^ quintile category. Municipal areas had a wide SES distribution, including facilities in all quintiles (Table 5).

**Table 5:** Distribution of facilities across socioeconomic status (SES) quintiles, by facility type, province, and distance from main town

**Categories**		**Total**	**SES Quintiles**^**2**^					**P**-**value**^**3**^
			**Poorest 20**%	**2**^**nd**^**quintile**	**3**^**rd**^**quintile**	**4**^**th**^**quintile**	**Least poor 20**%	
		**N**=**248**	**n**=**68**	**n**=**65**	**n**=**30**	**n**=**38**	**n**=**47**	
		%	%	%	%	%	%	
			[**95**% **CI**]	[**95**% **CI**]	[**95**% **CI**]	[**95**% **CI**]	[**95**% **CI**]	
All facilities %		100	20.4	20.3	20.3	20.9	18.2	
Facility type	Dispensaries	100	21.0	17.8	21.9	20.5	18.9	P = 0.16
	n=165		[11.1-36.2]	[10.8-27.8]	[12.8-34.8]	[9.9-37.8]	[10.8-30.8]	
	Health centers	100	17.2	33.0	12.0	23.0	14.9	
	n=83		[8.3-32.2]	[15.1-57.6]	[6.1-22.4]	[6.7-55.3]	[7.5-27.2]	
Province	Central	100	0	7.9	0	7.4	84.7	P<0.01**
	n=33			[1.0-41.1]		[1.3-31.8]	[42.0-97.7]	
	Coast	100	48.2	15.3	12.6	19.9	4.1	
	n=28		[13.2-85.1]	[6.4-32.0]	[3.1-39.6]	[3.0-66.5]	[0.5-27.5]	
	Eastern	100	37.4	17.9	21.5	10.4	12.7	
	n=33		[10.5-75.2]	[13.0-24.1]	[10.7-38.5]	[4.4-22.8]	[2.7-43.1]	
	North Eastern	100	64.9	32.0	0	0	3.0	
	n=25		[18.2-93.9]	[6.3-76.6]			[0.4-19.3]	
	Nyanza	100	27.1	8.9	38.3	25.7	0	
	n=25		[2.5-84.5]	[0.6-60.9]	[9.7-78.1]	[7.2-60.6]		
	Rift Valley	100	1.3	20.9	27.9	35.4	14.6	
	n=32		[0.1-12.7]	[5.6-54.0]	[14.3-47.1]	[12.0-68.7]	[5.6-33.1]	
	Western	100	33.9	51.6	8.4	6.2	0	
	n=33		[14.7-60.3]	[18.4-83.4]	[1.7-33.0]	[1.2-26.9]		
	Municipal^1^	100	25.7	12.9	10.0	16.3	35.1	
	n=39		[5.4-67.8]	[5.0-29.3]	[2.7-31.2]	[5.2-40.8]	[6.6-80.6]	
Remoteness	Near	100	19.8	20.7	17.4	16.6	25.5	P=0.50
	n=80		[7.6-42.7]	[11.8-33.8]	[7.1-36.6]	[7.5-32.6]	[16.4-37.3]	
	Average	100	24.3	18.6	17.7	25.5	13.8	
	n=140		[13.9-38.9]	[7.0-41.3]	[10.6-28.0]	[7.1-60.4]	[6.1-28.6]	
	Far	100	7.9	25.0	32.9	11.9	22.3	
	n=28		[1.9-28.0]	[11.4-46.2]	[14.1-59.4]	[0.8-68.7]	[8.6-46.7]	

^1^ Municipal areas included districts of Kilindini (Coast province), Nairobi West (Nairobi province), and Kisumu East (Nyanza province).

^2^ Weighted row percentage shown across SES quintiles. For example of dispensaries surveyed, 21.0% were in the poorest quintile of all facilities surveyed, and 18.9% were in the least poor quintile of facilities surveyed. The number of facilities per quintile is not equal as sampling weights are used in calculating the quintile cut-offs

^3^ Difference across groups tested using chi-squared test; *=p<0.05; **=p<0.01.

### Facility inputs

Of the eight facility input variables investigated, water, vaccine commodities, and family planning commodities were available in more than 75% of facilities (Table 1). Drugs had the lowest availability, with less than 20% of facilities having all tracer drugs in stock.

Chi-squared tests indicated significant variation in facility inputs across SES quintiles for electricity (p<0.01) only (Table 6). Pro-rich inequalities for electricity were also indicated by the equity ratio (3.16), and the concentration index (0.09, p<0.01). Availability of drugs had a significant concentration index (0.04, p<0.01) but was not statistically significant in the chi-squared analysis (Table 6).

**Table 6:** Availability of facility inputs and SES: Availability across SES quintiles, equity ratio, concentration index and indirectly standardized concentration index (*=p<0.05; **=p<0.01)

**Outcome**: **Facility inputs**	**SES Quintiles**					**P**- **value** (**chi**-**squared test of association by SES quintiles**)	**Equity ratio**	**Concentration index**^**1**^	**Indirectly standardized concentration index**^**1**^
	**Poorest 20**%	**2**^**nd**^**quintile**	**3**^**rd**^**quintile**	**4**^**th**^**quintile**	**Least poor 20**%				
	**n**=**68**	**n**=**65**	**n**=**30**	**n**=**38**	**n**=**47**				
	%	%	%	%	%				
	[**95**% **CI**]	[**95**% **CI**]	[**95**% **CI**]	[**95**% **CI**]	[**95**% **CI**]				
Equipment & infrastructure									
Equipment	61.1	52.5	80.0	48.1	69.7	P = 0.20	1.14	0.01	−0.02
N=248	[45.3-74.9]	[31.0-73.1]	[45.7-95.0]	[35.5-60.9]	[47.6-85.3]			P=0.76	P=0.49
Electricity	22.7	34.2	23.9	38.5	71.7	P <0.01**	3.16	0.09	0.07
N=247	[11.3-40.5]	[21.9-49.0]	[6.5-58.6]	[13.9-70.9]	[45.1-88.6]			P<0.01**	P<0.01**
Water	85.5	82.1	91.8	92.3	83.8	P = 0.70	0.98	0.01	−0.01
N=248	[72.1-93.0]	[39.1-97.0]	[75.7-97.5]	[58.2-99.1]	[41.3-97.5]			P-0.54	P=0.55
Commodities									
Drugs	16.1	6.0	22.1	21.1	31.7	P = 0.15	1.97	0.04	0.02
N=248	[7.9-30.0]	[3.1-11.5]	[6.8-52.8]	[10.8-37.0]	[15.3-54.4]			P<0.01**	P=0.10
Family planning	79.1	72.1	58.7	82.8	91.4	P = 0.20	1.16	0.02	0.01
N=247	[71.1-85.3]	[36.4-92.1]	[41.3-74.2]	[56.2-94.8]	[71.8-97.8]			P=0.48	P=0.83
Vaccines	70.3	72.9	76.6	84.7	83.2	P = 0.64	1.18	0.02	0.03
N=247	[53.2-83.2]	[45.1-89.8]	[32.5-95.7]	[48.4-97.0]	[61.5-93.9]			P=0.47	P=0.52
Staff									
Qualified staff	29.9	54.5	49.1	44.3	56.6	P = 0.32	1.89	0.03	0.03
N=248	[16.5-47.9]	[36.7-71.1]	[23.7-75.1]	[28.8-60.9]	[38.2-73.4]			P=0.08	P=0.04*
Support staff	64.1	68.4	57.2	50.2	78.3	P = 0.41	1.22	0.01	<−0.01
N=248	[44.6-79.9]	[37.2-88.7]	[39.0-73.7]	[36.0-64.4]	[49.8-92.9]			P=0.72	P=0.89

^1^ P-value for the concentration index shows whether the concentration index is significantly different from zero).

Indirectly standardized concentration indices showed that after adjusting for facility type, province and remoteness, SES remained a significant determinant of availability of electricity (0.07, p<0.01), but did not have a significant effect on drug availability. Although the chi-squared test and concentration index did not show a significant impact of SES on availability of qualified staff, the effect was significant based on the indirectly standardized concentration index (0.03, p=0.04) (Table 6).

### Services available

Family planning (97.4%), ITNs (84.4%), PMTCT (88.1%) and VCT/PITC (85.2%) were the most widely available services, with all antenatal care services offered every weekday in 74.6% of facilities. Laboratory (38.1%) and ART (26.6%) were least available, and less than half of facilities offered deliveries, or offered all childhood vaccines every day from Monday to Friday (Table 2).

Univariate analysis showed that availability of laboratory services differed across SES quintiles (p=0.02), with the equity ratio (3.43) and concentration index (0.05; p=0.01) also indicating pro-rich inequalities (Table 7).

**Table 7:** Availability of services and SES: Availability across SES quintiles, equity ratio, concentration index and indirectly standardized concentration index (*=p<0.05; **=p<0.01)

**Outcome**: **Availability of services**	**SES Quintiles**					**P**- **value** (**chi**-**squared test of association by SES quintiles**)	**Equity ratio**	**Concentration index**^**1**^	**INDIRECTLY standardized concentration index**^**1**^
	**Poorest 20**%	**2**^**nd**^**quintile**	**3**^**rd**^**quintile**	**4**^**th**^**quintile**	**Least poor 20**%				
	**n**=**68**	**n**=**65**	**n**=**30**	**n**=**38**	**n**=**47**				
	%	%	%	%	%				
	[**95**% **CI**]	[**95**% **CI**]	[**95**% **CI**]	[**95**% **CI**]	[**95**% **CI**]				
Laboratory	15.6	42.6	32.0	46.3	53.5	P = 0.02*	3.43	0.05	0.05
N=246	[8.2-27.7]	[23.3-64.5]	[12.4-61.1]	[31.8-61.5]	[35.5-70.6]			P=0.01*	P=0.01*
All childhood immunizations every weekday	47.6	59.7	42.3	31.5	47.7	P = 0.22	1.00	−0.02	−0.03
N=247	[30.9-64.7]	[33.9-81.1]	[13.8-77.1]	[16.8-51.1]	[26.7-69.5]			P=0.15	P=0.20
All antenatal care every weekday	83.3	73.9	63.5	67.0	87.4	P = 0.40	1.05	<0.01	<0.01
N=246	[62.5-93.7]	[45.6-90.5]	[18.1-93.2]	[53.6-78.1]	[71.7-95.0]			P=0.89	P=0.97
Family planning	92.9	100	100	97.3	96.4	P = 0.31	1.04	<−0.01	<−0.01
N=247	[81.5-97.5]			[81.3-99.7]	[83.7-99.3]			P=0.81	P=0.35
Delivery	60.5	57.5	36.6	48.3	42.1	P = 0.57	0.70	−0.37	<−0.01
N=247	[39.2-78.4]	[25.8-84.1]	[12.8-69.4]	[35.9-60.9]	[19.0-69.4]			P=0.28	P=0.87
VCT/PITC	88.1	66.7	87.0	97.3	86.9	P=0.05	0.99	0.15	0.04
N=247	[79.5-93.5]	[46.2-82.4]	[62.3-96.4]	[81.3-99.7]	[67.5-95.5]			P=0.42	P=0.03*
PMTCT	91.7	83.4	85.2	90.1	90.7	P = 0.76	0.99	<0.01	0.01
N=240	[80.2-96.8]	[37.4-97.7]	[43.2-97.7]	[66.9-97.6]	[73.8-97.1]			P=0.82	P=0.74
ART	23.1	34.0	29.2	25.6	20.3	P = 0.62	0.88	<−0.01	<−0.01
N=247	[12.4-38.9]	[20.9-50.1]	[10.5-59.2]	[15.6-39.0]	[12.8-30.7]			P=0.65	P=0.99
ITN	81.6	74.7	99.7	82.7	82.9	P = 0.39	1.02	<−0.01	−0.02
N=247	[68.5-90.0]	[37.0-93.7]	[98.4-99.9]	[35.3-97.7]	[48.5-96.1]			P=0.93	P=0.60
Outreach	35.9	45.5	56.5	46.5	38.8	P = 0.56	1.08	<−0.01	0.03
N=247	[26.4-46.7]	[32.1-59.6]	[22.3-85.4]	[30.9-62.8]	[15.4-68.9]			P=0.91	P=0.08

^1^ P-value for the concentration index shows whether the concentration index is significantly different from zero.

After adjustment for facility type, province and remoteness, SES remained a significant determinant of availability of laboratory services (0.05, p=0.01). Although the chi-squared test and concentration index did indicate a significant impact of SES on VCT/PITC services, the effect was significant based on the indirectly standardized concentration index (0.04, p=0.03) (Table 7). No other significant associations were identified between SES and service availability.

## Discussion

### Limitations

There are five caveats with the data used, for which the impact on analysis of inequality is unclear. First, the administrative area of location used to calculate the SES of the facility is a proxy measure for catchment area, and may not accurately measure the SES of the catchment area for facility users or the area in which most facility users reside. However, the median area of 16km^2^ for the location appears quite consistent with typical distances users travel to government facilities (5-6km) [31,32]. Second, SES data are from relatively old surveys (1997/99) [20], and the relative SES of facility locations may have changed overtime. More recent SES data were available from a 2005–6 household consumption survey [35] but the data were only disaggregated to the district level using the old boundaries for 69 districts, and therefore were of limited value in providing information on the specific SES characteristics of individual health facilities.

Third, the creation of input availability indicators involved some decisions on cut offs (at least 4 qualified staff, at least 2 support staff, and at least 15 out of 18 equipment items), which are somewhat arbitrary. We tested the robustness of our findings to variations in these cut-offs, but found no changes in the significance of the univariate results^e^. Fourth, the equipment, drug, family planning and vaccine indicators imply that all individual commodities in these indicators are of equal importance, whereas in reality, for example, the lack of certain drugs on the tracer list may be much more significant for health outcomes than the lack of others.

Finally we were unable to conduct the survey at 24 of the facilities originally selected (4.6% of the sampling frame of facilities in selected districts) because they were not operational, not officially recognized by the government, or did not have any qualified staff. These exclusion criteria were used because the survey was conducted as the baseline for the evaluation of a new health financing mechanism for which only fully functioning facilities were eligible. Therefore, we are likely to have excluded some of the poorest performing facilities; if these were concentrated in poorer areas, this could have led to an under-estimation of inequalities. However, of the 24 dispensaries excluded, poverty data were available for 12 facilities^f^, and these were relatively evenly distributed across the SES quintiles of surveyed facilities (2 each in poorest and least poor quintiles, 3 in 2^nd^ quintile and 5 in 3^rd^ quintile).

In addition there are 3 limitations to the study design, which may mean that inequalities are underestimated in this analysis. First, our approach does not consider areas that had no facility at all within reasonable reach. Again it is likely that these areas are relatively poor and their populations therefore have no access to services. Secondly, we only included health centers and dispensaries, excluding public hospitals, as the expectation of inputs and services in the latter is markedly different. However, many hospitals do offer similar outpatient services to the smaller facilities, and as they are generally located in larger centers, which are likely to be relatively well off, this may have led us to underestimate inequalities in primary care provision.

Thirdly the analysis focused exclusively on government facilities, which can be considered appropriate given their use of public resources. Faith based facilities are important care providers in some areas of the country, and if the SES distribution and pattern of service and input availability differed between government and faith based facilities, their inclusion could affect overall inequity. However, the Kenya Service Provision Assessment indicated that relative input and service availability between faith based and public facilities is mixed across indicators, with no clear pattern overall. [36,37]. Moreover, based on poverty data of the locations of faith based facilities, we did not find evidence that they were concentrated in poorer areas than government facilities. The proportion of the population above the poverty line was 27.9% in the poorest 20% of government facilities compared with 29.7% in the poorest 20% of faith based facilities, and 70.6% in the richest 20% of government facilities compared with 75.5% in the richest 20% of faith based facilities.

### Key findings

Addressing inequity in access to health care is a key concern in Kenya and is documented as a policy priority. This paper assessed equity of facility input and service availability in public primary health facilities across the country. For most input and availability indicators in primary health care facilities, we found no indication of variation by SES of the facility’s location. This could be considered an encouraging finding, indicating that availability of care is relatively equitable across this diverse country.

In addition, availability of some inputs and services was generally high. Close to 90% of facilities had a functioning water supply and almost all offered family planning services. More than 80% offered VCT/PITC, PMTCT and ITN services. However, the lack of inequality does not imply that availability of inputs and services were invariably high. For example, only around three quarters of facilities had all family planning or all vaccine commodities in stock, or offered antenatal care services every weekday. Only around two thirds had at least 15 of 18 key equipment items available, or had at least 2 support staff. Under half offered delivery services, all childhood immunizations every weekday, outreach services, or had at least 4 qualified staff. Only a little more than one-third had laboratory services or electricity available, and less than 20% had all drugs on the tracer list in stock. These results were similar to those for government facilities from the Kenya Service Provision Assessment Survey (KSPA) conducted in the same year for electricity and family planning, though we found higher availability for water supply, perhaps reflecting a broader definition that was used in our analysis [36].

For specific indicators, we found clear evidence of pro-rich inequalities for electricity and laboratory services, and some indications of pro-rich inequalities for availability of drugs and qualified staff. Drug availability and qualified staff are central inputs into both technical quality of care and demand for facility services, and laboratories are important for accurate disease diagnostics. Electricity is important for enhancing quality of care to patients at the facility in several ways. Although fridges can be run on gas cylinders where there is no electricity (79% of facilities without functioning electricity had functioning fridges), electric lighting is useful for providing care at night and for security, and in some cases is required for equipment such as microscopes, autoclaves and computers. It seems plausible that electricity and laboratory services would be more available in better off areas because they often rely on local funding from user fees, which better-off clients are more willing and able to pay. Electricity bills for health centers and dispensaries generally have to be paid from facility revenues, and laboratories are often established partly as an income-generating activity for the facility, with fees per service provided [18]. However, we did not find pro-rich inequalities in other locally funded inputs such as water and support staff. Kenya is currently introducing a new system of financing health centers and dispensaries called the Health Sector Services Fund (HSSF), which transfers money directly into facility bank accounts to be managed by the health facility committee, and used for a range of operational expenses [18]. It is possible that this additional funding controlled at the facility level will allow more facilities in relatively poor areas to improve availability of electricity and laboratory services in future, and enable all facilities to expand their outreach activities, support staff and equipment availability.

## Conclusions

We have shown how local area poverty data can be combined with national health facility surveys, providing a simple tool for policy makers to assess the equity of health care service availability. While there are some limitations with the data, the general indications in Kenya were that there was little evidence of marked inequalities of input and service availability, although we did identify pro-rich inequalities in the availability of electricity and laboratory services, and possibly for drug supply, and qualified staff. While there was limited evidence of inequalities, it should be noted that availability of some inputs and services was universally poor in areas of all SES levels. Moreover, it is important to note that variations in service availability are only one determinant of inequality in health care utilization and outcomes. The latter are also highly dependent on health financing systems, the knowledge of community members, and the quality of care provided, which depends on health worker performance as well as input availability.

### Endnotes

^a^ For sampling purposes the districts were defined based on the boundaries of the 149 districts that the Ministry of Public Health and Sanitation (MOPHS) was working with as of January 2009, although several districts have since been sub-divided. All districts were included in the sampling frame with the exception of Siaya and Kisumu West, both in Nyanza Province, and Kilifi in Coast Province because the intensity of Kenya Medical Research Institute (KEMRI) research activities in these districts and Millennium Village activities in Siaya meant that the majority of health facilities in these districts were highly atypical.

^b^ The sample size was based on the needs of a before and after evaluation of a new health financing mechanism, for which this survey represented the baseline.

^c^ OANDA average exchange rate, 1997, http://www.oanda.com

^d^ WMS data was missing for some parts of North Eastern Province (Mandera, Wajir and Garissa districts), and Tana River district in Coast Province, so for these areas values were imputed using estimations based on data from the rest of the country.

^e^ We varied the cut off on equipment from 10 to 16 items, on qualified staff from 2 to 6 staff, and on support staff from 1 to 3 staff.

^f^ Poverty data were not available for the other excluded facilities because they were not included in the national facility database; in many cases this was likely to be because they were newly established.

## Abbreviations

ANC: Antenatal care; ART: Anti-Retroviral Treatment; BCG: Bacillus Calmette Guerin (TB vaccine); BP: Blood Pressure; DHMT: District Health Management Team; DPT: Diphtheria Pertussis (Whooping cough) and Tetanus vaccine; HepB: Hepatitis B; Hib: Haemophilus influenzae type B vaccine; ITNs: Insecticide Treated Nets; KEMRI: Kenya Medical Research Institute; KES: Kenyan Shillings; KSPA: Kenya Service Provision Assessment Survey; MOPHS: Ministry of Public Health and Sanitation of Kenya; OPV: Oral Polio Vaccine; ORS: Oral Re-hydration Salts; PITC: Provider Initiated Testing and Counseling; PMTCT: Prevention of Mother to Child Transmission; SES: Socioeconomic status; USD: United States Dollars; VCT: Voluntary Testing and Counseling; WMS: Welfare Monitoring Surveys.

## Competing interests

The authors declare that there are no competing interests.

## Authors’ contributions

CM and CG were involved in the conception and design of the study. AO, GF, CM, and CG supported the design of data collection tools and sampling calculation. AO, EW, MT, CM, and CG participated in data collection. MT, AN, TE, GF, CM, and CG supported the analysis and write up. All authors read and approved the final manuscript.
